# Data on the genome analysis of the wild edible mushroom, *Russula griseocarnosa*

**DOI:** 10.1016/j.dib.2019.104295

**Published:** 2019-07-19

**Authors:** Fei Yu, Junfeng Liang

**Affiliations:** Key Laboratory of State Forestry Administration on Tropical Forestry Research, Research Institute of Tropical Forestry, Chinese Academy of Forestry, Guangzhou 510520, China

**Keywords:** *Russula griseocarnosa*, Ectomycorrhizal fungus, Whole genome, Genome annotation

## Abstract

In the present article, we report data on the whole genome sequence of a wild edible and medicinal ectomycorrhizal fungus *Russula griseocarnosa*. The *R. griseocarnosa* genome consists of 64.81 Mb with a GC-pair content of 49.41%. The genome assembly consists of 471 scaffolds and 16128 coding protein genes. The coding protein genes was annotated in different databases (GO, KEGG and CAZys), respectively. The whole genome sequence and functional annotation provide important information for ectomycorrhizal fungus, which can be used as a basis for cultivation and breeding of *R. griseocarnosa*. The Whole Genome project of *Russula griseocarnosa* has been deposited at DDBJ/ENA/GenBank under the accession RMVF00000000. The version described is RMVF01000000. To further interpretation of the data provided in this article, please refer to the research article ‘Whole genome sequencing and genome annotation of the wild edible mushroom, *Russula griseocarnosa*’ [1].

**Table:** Specifications Table

Subject area	Biology
More specific subject area	Microbiology, Genomics
Type of data	Table, figures
How data was acquired	PacBio RS and Illumina Hiseq X-Ten sequencing
Data format	Annotated and comparative analyzed
Experimental factors	The fruiting body samples were obtained and quickly frozen in liquid nitrogen before stored in a −80 °C freezer. Total DNA of fruiting body was extracted immediately.
Experimental features	DNA Sequencing was performed by using PacBio RS and Illumina Hiseq X-Ten, genome assembly, annotation and analysis were carried out.
Data source location	The fruiting bodies of *Russula griseocarnosa* were collected from Linjing Town, Teng County, Guangxi Province, China (2 Jun. 2017) (23.15 N, 110.66 E)
Data accessibility	The whole genome sequence of *Russula griseocarnosa* has been deposited at DDBJ/ENA/GenBank under the accession RMVF00000000. The version described is RMVF01000000. The BioSample, BioProject and SRA accession number are SAMN09602224, PRJNA479704 and SRP153002, respectively.
Related research article	F. Yu, J. Song, J.F. Liang, S.K. Wang, J.K. Lu, Whole genome sequencing and genome annotation of the wild edible mushroom, *Russula griseocarnosa*. Genomics. (2019) in press [1]https://doi:10.1016/j.ygeno.2019.04.012.

**Table:** 

**Value of the data** • The first genome under the genus *Russula* to be reported. • The data provide valuable information of the potential function and gene expression mechanisms about ectomycorrhizal fungus *Russula griseocarnosa.* • The CAZymes of *Russula griseocarnosa* confirms the adaptation to symbiosis, and reveals the strategy for host interaction.

## 1 Data

**Fig. 1:**
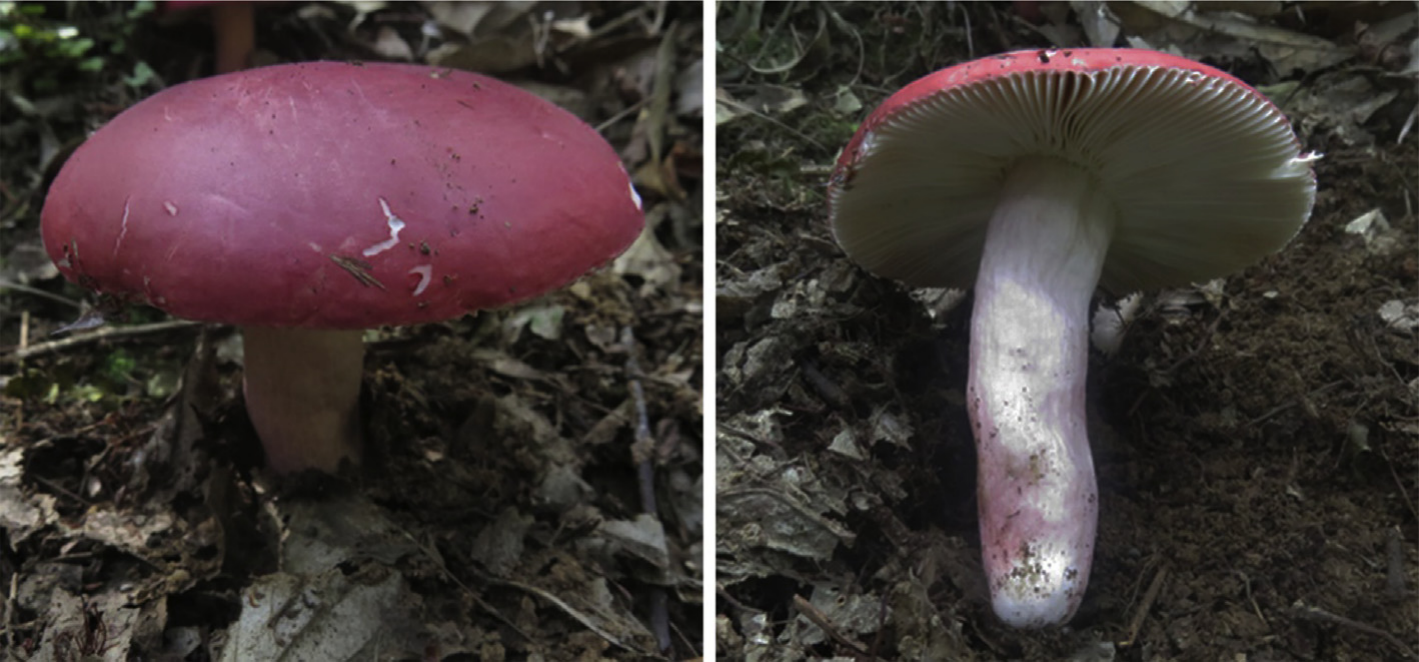
Fruiting bodies of *Russula griseocarnosa*.

**Fig. 2:**
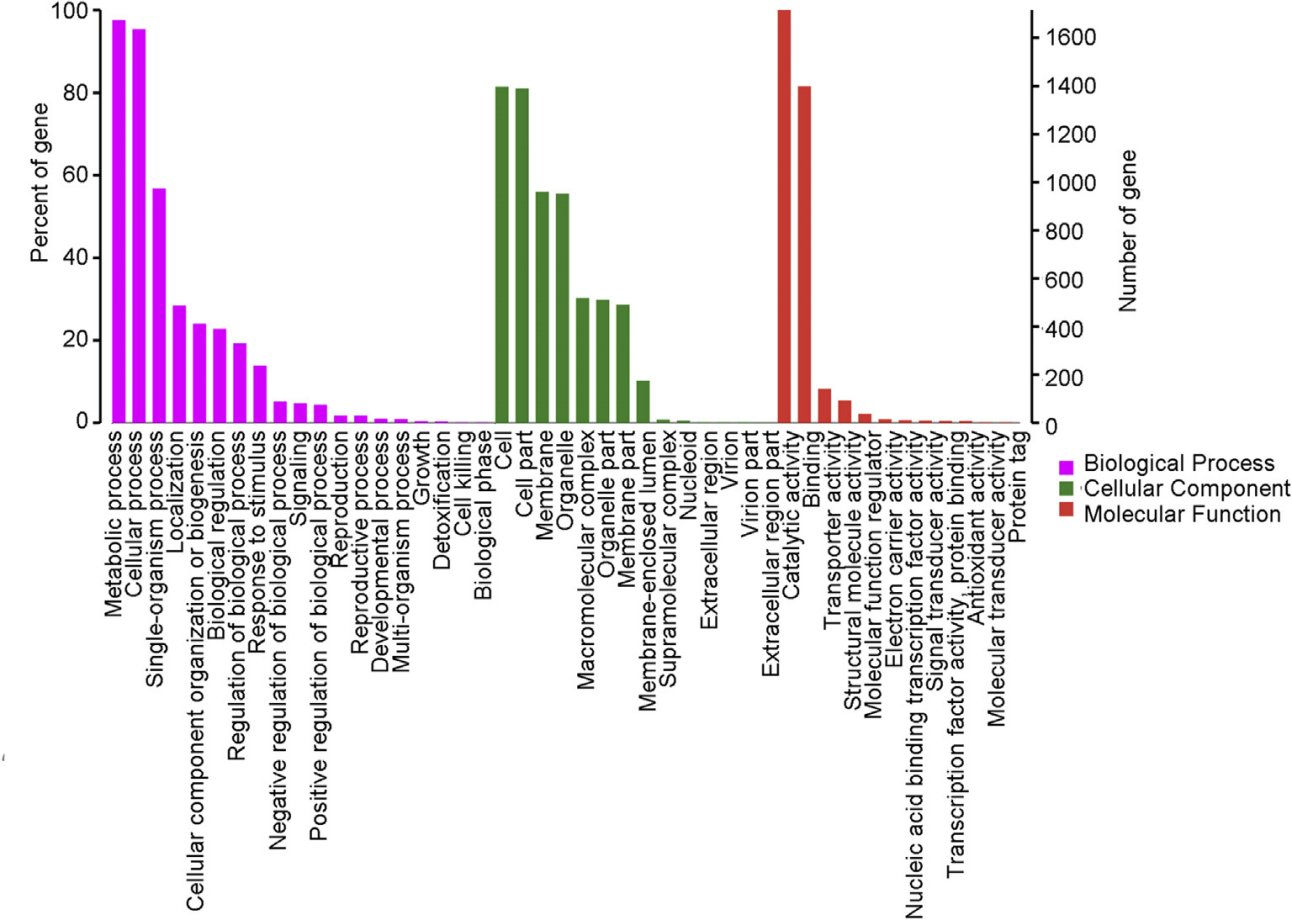
The Gene Ontology (GO) function annotation of *Russula griseocarnosa*.

**Fig. 3:**
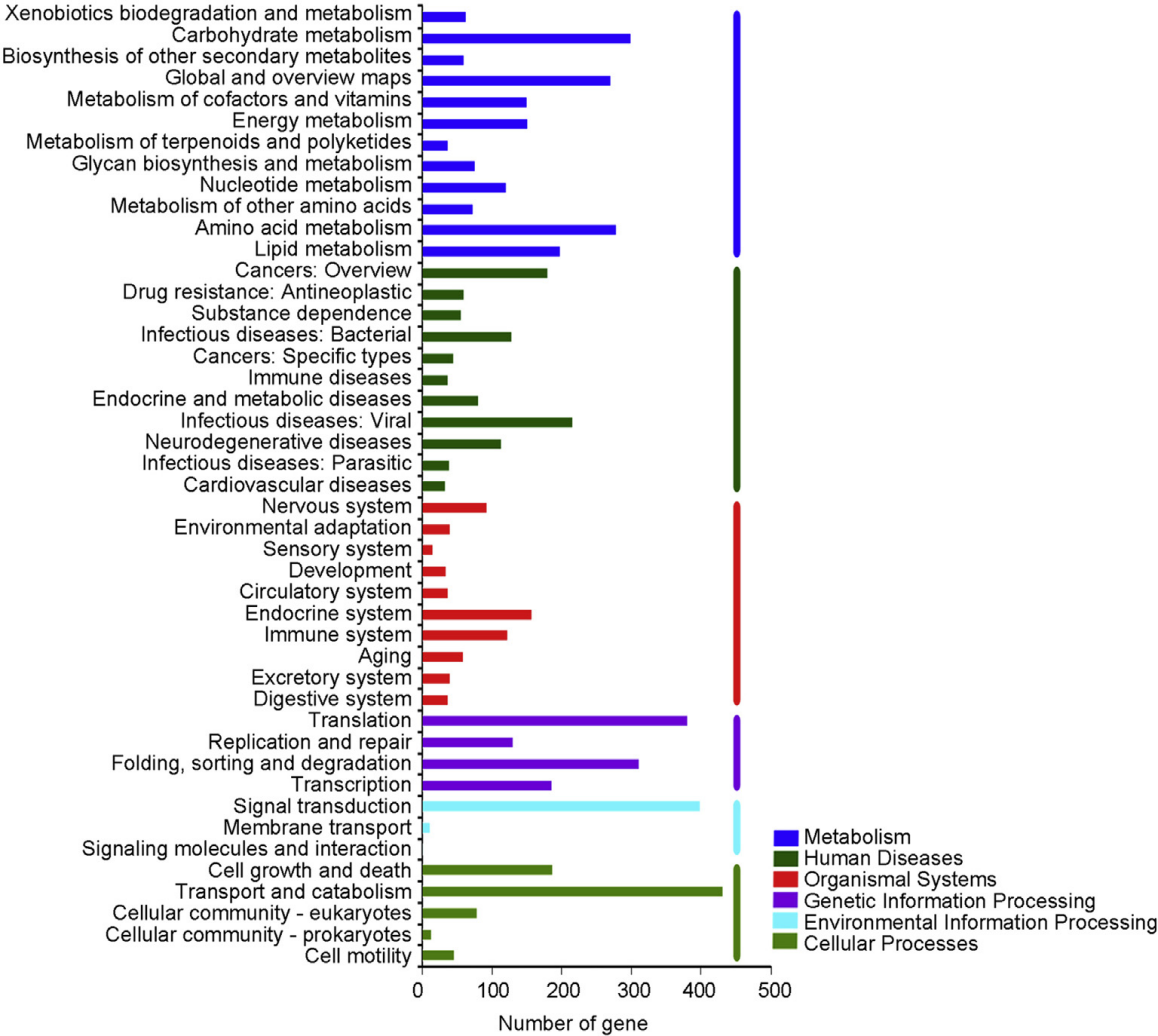
The KEGG function annotation of *Russula griseocarnosa*.

*Russula griseocarnosa* (Fig. 1) is a wild edible and medicinal ectomycorrhizal fungus that is native to southern China. The resulting draft genome of *R. griseocarnosa* present the 64.81 Mb in size with a G+C content of 49.41%. The genome sequence was assembly with 471 scaffolds and 16128 coding protein genes [1]. The data illustrated in Fig. 2 show the Gene Ontology (GO) distribution of the protein coding genes and Fig. 3 gives a complete overview of the KEGG pathway. According comparative analysis, The GO annotations of *Russula griseocarnosa* genes were similar with *Agaricus bisporus* [2] in “Localization”, “Biological regulation”, and “Regulation of biological process”, and fewer numbers than that of *Laccaria bicolor* [1], [3]. Compared with KEGG metabolic annotations, the most genes of *Russula griseocarnosa* pathways was not significantly in *Laccaria bicolor* and *Agaricus bisporus*, but *R. griseocarnosa* had less genes in "Tryptophan metabolism" and "Starch and sucrose metabolism" pathways [1].

**Fig. 4:**
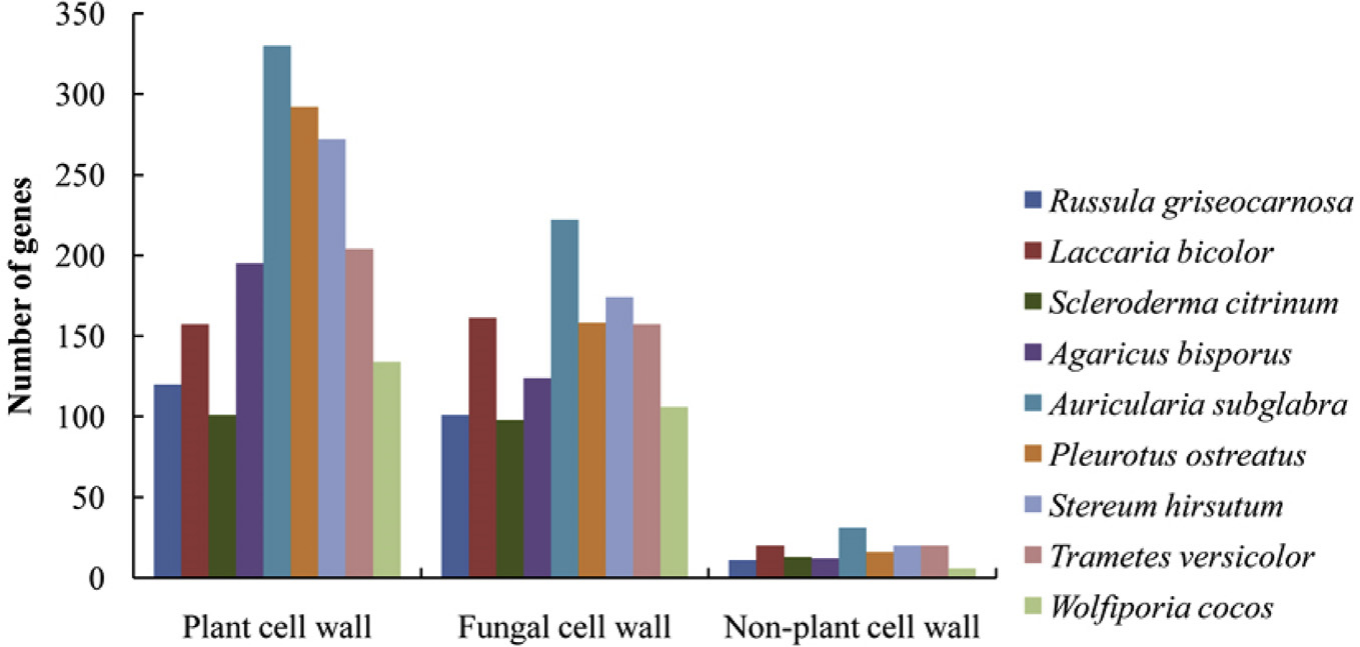
Comparison of CAZys associated with cell wall degradation.

The CAZymes coding genes of *R. griseocarnosa* encode enzymes involved in the degradation of plant cell wall polysaccharides, non-plant polysaccharides (for example, animal and bacterial polysaccharides) and fungal cell wall (Fig. 4). The CAZymes coding genes of *R. griseocarnosa* was similar to the symbiotic fungal species *Scleroderma citrinum* [4] in non-plant polysaccharides degradation and fungal cell wall degradation, and higer number of plant cell wall polysaccharides degradation. The plant cell wall polysaccharides degradation associated with cellulose degrading enzymes (GH6, GH7, GH44 and GH45), hemicellulose-degrading enzymes (GH10, GH11 and GH115) and pectin-degrading enzymes (GH43, GH51, GH78, GH93, PL1, PL3, and PL4) were absent in *Russula griseocarnosa*, *Laccaria bicolor*, and *Scleroderma citrinum* genomes [1].

## 2 Experimental design, materials and methods

### 2.1 Fungal material

Fruiting bodies of *R. griseocarnosa* were collected from Linjing Town, Teng County, Guangxi Province, China in 2017. The fruiting body samples was frozen in liquid nitrogen and stored at −80 °C freezer until DNA extract.

### 2.2 DNA extraction and sequencing

Genomic DNA was extracted using the Omega Fungal DNA Kit D3390-02. Quality of DNA was determined using TBS-380 fluorometer (Turner BioSystems Inc., Sunnyvale, CA). The concentration of at least 20 mg/L (OD260/280 = 1.8–2.0).

*R. griseocarnosa* genome was sequenced using Illumina HiSeq X-ten sequencing and PacBio RS sequencing at Shanghai Majorbio Bio-pharm Biotechnology Co., Ltd, China. Paired-end libraries with 300 bp inserts were constructed in Illumina HiSeq X-ten sequencing. 8-10k insert shotgun libraries were generated in Pacific Biosciences RS sequencing.

### 2.3 Genome assembly and annotation

The genome sequence was assembled as follows: (1) PacBio long reads were corrected and assembled by Canu (v1.7) [5]; (2) Illumina reads corrected and used for scaffolding by SOAPdenovo (v2.04). Fill the gaps using GapCloser (v1.12) package; and (3) PacBio reads were modified based on Illumina reads. The final assembly produced a circular genome sequence without gaps.

Protein coding sequences were predicted using the automated pipeline MAKER2 (v2.31.9) [6]. It combining data for mRNAs, proteins, the ab initio predictions of SNAP [7] and GeneMark-ES (v2.3a) [8].

The predicted protein coding sequences was annotated in Gene Ontology (GO) and Kyoto Encyclopedia of Genes and Genomes (KEGG) database using Blastp (v2.3.0). The Carbohydrate-active enzymes (CAZymes) were performed using blastp (cut off e-value≤1e−5) at http://www.cazy.org/ [9].
